# Gene expression plasticity across hosts of an invasive scale insect species

**DOI:** 10.1371/journal.pone.0176956

**Published:** 2017-05-04

**Authors:** Nicholas Christodoulides, Alex R. Van Dam, Daniel A. Peterson, Rasmus John Normand Frandsen, Uffe Hasbro Mortensen, Bent Petersen, Simon Rasmussen, Benjamin B. Normark, Nate B. Hardy

**Affiliations:** 1 Department of Entomology and Plant Pathology, Auburn University, Auburn, Alabama, United States of America; 2 Biosynthetic Pathways Engineering, Department of Bioengineering, Denmark Technical University, Søltofts plads, Lyngby, Denmark; 3 Department of Biology and Graduate Program in Organismic and Evolutionary Biology, University of Massachusetts, Amherst, Massachusetts, United States of America; 4 Center for Biological Sequence Analysis, Department of Systems Biology, Technical University of Denmark, Kemitorvet, Lyngby, Denmark; Natural Resources Canada, CANADA

## Abstract

For plant-eating insects, we still have only a nascent understanding of the genetic basis of host-use promiscuity. Here, to improve that situation, we investigated host-induced gene expression plasticity in the invasive lobate lac scale insect, *Paratachardina pseudolobata* (Hemiptera: Keriidae). We were particularly interested in the differential expression of detoxification and effector genes, which are thought to be critical for overcoming a plant’s chemical defenses. We collected RNA samples from *P*. *pseudolobata* on three different host plant species, assembled transcriptomes *de novo*, and identified transcripts with significant host-induced gene expression changes. Gene expression plasticity was pervasive, but the expression of most detoxification and effector genes was insensitive to the host environment. Nevertheless, some types of detoxification genes were more differentially expressed than expected by chance. Moreover, we found evidence of a trade-off between expression of genes involved in primary and secondary metabolism; hosts that induced lower expression of genes for detoxification induced higher expression of genes for growth. Our findings are largely consonant with those of several recently published studies of other plant-eating insect species. Thus, across plant-eating insect species, there may be a common set of gene expression changes that enable host-use promiscuity.

## Introduction

The overwhelming majority of plant-eating insect species are host-specialists that consume only one or a few closely-related plant species [1, 2]. But plant-eating insect communities predictably include a few species that are host-generalists, some of which can have extremely broad diets [2, 3]. These generalists are critical elements in terrestrial food webs; their high degree of ecological connectedness is a critical part of what makes ecosystems resilient and many specialist niches possible [4]. Generalists are also enriched in invasive and pest insect faunas, which gives them more economic weight than their species richness might suggest [5]. What genetic systems enable host-use promiscuity in plant eating insects? Some key host-use genes and proteins have been identified [6], and in the last few years there has been a number of studies examining how gene expression varies across hosts [7, 8]. Nevertheless, at this stage, few plant-eating insect species and host interactions have been studied, and we still know little with confidence about the genetic architecture of generalism in plant-eating insects.

We typically assume that the main limiters on plant-eating insect diets are plant defenses, and that a plant-eating insect’s diet breadth is determined by how many of those defenses it can overcome [9]. Researchers have paid particular attention to a plant-eating insect’s ability to detoxify plant defensive chemicals with a core suite of enzymes in a handful of large gene families: carboxylesterases, UDP-glycosyltransferases, glutathione-S-transferases, cytochrome oxygenase P450s, glutathione peroxidases, and ATP-binding cassette transporters (ABC transporters) [9, 10, 11]. However, this list may be too restrictive. For example, zinc-binding dehydrogenases have been implicated in plant-defense compensation in peach aphids [12]. And recently, researchers have come to recognize that the fitness of some plant-eating insects on their host plants depends on effectors, that is, secreted macromolecules that attenuate a plant’s inducible defenses [13, 14]. Disrupting a host plant’s inducible defenses may be especially important for phloem-sap-sucking insects—such as aphids, whiteflies and scale insects—as they have close and persistent contact with their hosts [15]. Less attention has been paid to how factors other than plant defense, such as nutrition, could limit a plant-eating insect’s diet, but non-defensive factors may be important.

The recent spate of comparative transcriptomics studies indicates that broad diets are, in fact, facilitated by differential expression of detoxification genes [8, 16, 17]. However, those studies also indicate that detoxification genes are only a small part of the picture. Most of the significant host-induced changes in gene expression do not appear to be related to detoxification. For example, researchers have found consistent host-dependent expression of many genes involved in primary metabolism [8, 18]. Furthermore, some studies have recovered evidence of a trade-off between expression of genes for detoxification and primary metabolism [7, 18]; for some plant-eating insects, increased investment in the expression of detoxification genes may come at the cost of reduced investment in growth and reproduction. But this is not the case for all insects. In the silverleaf whitefly, *Bemisia tabaci*, we find just the opposite: expression of genes for detoxification and primary metabolism rise and fall together [19]. Thus, the generality of this trade-off is not clear. Also unclear is the extent to which expression of effector genes varies across host plant species (but see [16] for evidence that it is important in host-use adaptation). Effector genes are involved in many biological processes and have many molecular functions. Consequently, there is no effector category in standard gene ontologies, and researchers would not detect enriched differential expression of effectors across hosts using standard enrichment analyses. Furthermore, with one exception [20], the published RNAseq-based differential expression analyses of insect diet variation have studied species with transient connections with their host plants (at least in comparison to sap-sucking insects), and might not be expected to strongly manipulate their host’s induced defenses.

The lobate lac scale, *Paratachardina pseudolobata* (Hemiptera: Coccoidea: Kerriidae), is a good example of host-generalism (polyphagy) in plant-eating insects, and why it is worth studying. Thought to be native to Southeast Asia [21], the lobate lac scale is invasive and widespread in nearly all areas of Florida. It has been recorded from more than 307 plant species, including several of ecological and economic importance. Females are parthenogenic and sessile phloem feeders. They form dense aggregations on branches and stems, and excrete sugar-rich waste that feeds harmful sooty molds [22]. Some native plant species seem to be particularly vulnerable to lobate lac scale infection, including wax myrtle (*Myrica cerifera*), a favorite nesting area for many water-wading birds [23]. Controlling lobate lac scale populations has proven difficult; natural enemies are lacking in Florida, and thick resinous wax protects females from many insecticides [21].

In this study we investigated the genetic architecture of host-generalism in *Paratachardina pseudolobata*. Specifically, we examined gene expression variation across field-collected samples taken from multiple locations and hosts in southern Florida. We were particularly interested in 1) determining how expression of effector genes depends on host species, 2) confirming the importance of the differential expression of detoxification genes across hosts, and 3) assessing if there are trade-offs between the expression of genes involved in primary metabolism and those involved in plant defense compensation.

## Materials and methods

### Sample collection

*P*. *pseudolobata* samples of whole, adult females were collected from four locations in southern Florida (Table 1). No specific permissions were required for these collections, and the insects are not protected or endangered species. Each sample was made up of about twenty specimens. A total of nine samples were taken from three host plants: two samples from *Tetrazygia bicolor* (Melastomataceae), three samples from *Myrsine cubana* (Myrsinaceae), and four samples from *Psychotria nervosa* (Rubiaceae). Specimens were plucked from their host plants and immediately homogenized in tubes filled with Trizol reagent. The ~20 specimens in each tube were pooled.

**Table 1:** Samples of lobate lac scales.

Location	Host species
Hugh Taylor Birch State Park, Ft Lauderdale, FL	*Psychotria nervosa*
IFAS Fort Lauderdale Research & Education Center, Davie, FL	*Tetrazygia bicolor*
IFAS Fort Lauderdale Research & Education Center, Davie, FL	*Myrsine cubana*
IFAS Fort Lauderdale Research & Education Center, Davie, FL	*Psychotria nervosa*
IFAS Fort Lauderdale Research & Education Center, Davie, FL	*Psychotria nervosa*
Navy Wells Pineland Preserve, Homestead, FL	*Tetrazygia bicolor*
Navy Wells Pineland Preserve, Homestead, FL	*Myrsine cubana*
Tree Tops Park, Davie, FL	*Myrsine cubana*
Tree Tops Park, Davie, FL	*Psychotria nervosa*

### RNA extraction, library, preparation, and sequencing

Each RNA sample was homogenized using the Fast Prep FP120 Homogenizer Cell Disrupter (Thermo Fisher) for three pulses of 20 seconds using small steel beads. Then, 700μL of each sample was transferred to a fresh centrifuge tube with 200μL of chloroform and shaken vigorously for 15 seconds and then incubated on ice for 20 minutes, shaking every 10 minutes. Samples were then centrifuged for 15 minutes at 14,000rpm at 4°C. Approximately 400μL of each sample was added to an equal amount of 100% EtOH at 4°C. This mixture was then passed through the standard protocol of the TRIzol^®^ Plus RNA Purification Kit (Thermo Fisher). RNA yields and qualities were assessed using a Qubit fluorometer and an Agilent 2100 Bioanalyzer at the Denmark Technical University Multi-Assay Core (DMAC). CDNA Libraries were prepared at the University of California Davis Genome Center DNA Technologies Core. Samples were poly-A tailed, normalized, and pooled using Illumina TruSeq adapters. Samples were then loaded across three lanes of a single flow cell and sequenced in 100 base paired-end reads using an Illumina HiSeq3000.

### Quality control and transcriptome assembly

We removed adapters and low-quality sequences (quality score cutoff = 25) from the raw sequence reads using Trimmomatic 0.35 [24] and discarded reads less than 36 bp long. We then merged and normalized the quality-trimmed reads by kmer coverage and length using Khmer [25]. We assembled normalized reads into transcriptomes *de novo* using both Velvet 1.2.08 and Trinity [26, 27]. We constructed the Trinity assembly using default parameters (kmer = 25). We constructed eight Velvet assemblies, each with scaffolding enabled, and a minimum transcript length of 200bp, but with eight different kmer lengths: 27, 29, 31, 33, 35, 43, 53, 63. We then combined all assemblies from both programs into a single Fasta file and passed it through the EvidentialGene pipeline [28] to cluster sequences based on similarity, and find the best consensus transcripts for a final assembly. We assessed the quality of the assembly with BUSCO and Transrate [29, 30]. The Transrate assembly score was 0.30, which is considered a passing score based on the number of input reads mapping to the assembly. BUSCO found that 89% of highly conserved arthropod sequences were present as single-copy or duplicated transcripts in the assembly, suggesting that it is mostly complete. For annotation and gene ontology assignments we passed the assembly through Annocript, BLASTing against the Uniref90, SwissProt and Conserved Domain databases with an e-value cut-off value of 1e-5 [31, 32]. We used these annotations for all subsequent analysis.

### Differential expression and GO enrichment analysis

We quantified the number of each transcript represented in the quality-trimmed read libraries by mapping them to the final assembly using eXpress and Bowtie2 [33, 34]. To determine how gene expression in lobate lac scale populations varied across host plant species, we first created a matrix of transcript counts using Trinity scripts. Next, we used the Bioconductor package Limma to convert the transcript counts to log-counts per million and the voom function to model the mean-variance relationship with precision weights across the three host plant species with three pair-wise comparisons [35]. We excluded low-abundance transcripts from the analysis. We used a false discovery rate (FDR) cutoff value of 0.05 to classify transcripts as differentially expressed, and made an expression heat map via Trinity scripts. We also used TopGO with gene ontology (GO) annotations provided by Annocript to perform GO term enrichment analysis [36]. TopGO categorizes differentially expressed transcripts under GO terms relating to general biological processes and molecular functions. We summarized these results with REVIGO [37].

We then looked specifically at the expression of detoxification and effector genes across hosts. For detoxification genes, we examined transcripts which had been annotated as carboxylesterases, UDP-glycosyltransferases, glutathione-S-transferases, cytochrome oxygenase P450s, glutathione peroxidases, and ABC transporters. Investigation of insect effector genes was less straightforward, as they have only recently been characterized, fall into a variety of gene ontology classes, and are unlikely to be in curated protein databases. We began with a list of 67 candidate effector genes in the aphids *Acrythosiphon pisum* and *Myzus persicae* provided by [38]. We then extended this list to include 20 predicted effectors from aphid salivary gland transcriptomes [10]. We collated sequences for the genes in this list and identified lobate lac scale homologs in our assembly using the tblastx function in BLAST+ with an e-value cutoff of 0.00001.

## Results

### Assembly and annotation

Sequencing generated ~120 million paired-end reads per library. The final, consensus assembly contained 113,670 transcripts, with a contig N50 of 1055 base pairs and a longest contig length of 33,906 base pairs. Annotation yielded 82,024 transcripts with at least one database hit. In comparison to published transcriptomes, the lobate lac transcriptome is most similar to those of the two-spotted spider mite, *Tetranychus urticae*, and the pea aphid, *Acrythosiphon pisum*. The most diverse genes in the assembly included sugar transporters, protein kinases, Ras family proteins, ubiquitins, and cytochrome P450s. Transcripts annotated as detoxification genes in the assembly included 354 cytochrome P450 oxygenases, 12 carboxylesterases, 96 glutathione S-transferases, 78 UDP-glycosyltransferases, 10 glutathione peroxidases, and 646 ABC transporter transcripts. Additionally, we found 1015 transcripts closely matching candidate aphid effectors.

### Differential expression

**Fig 1:**
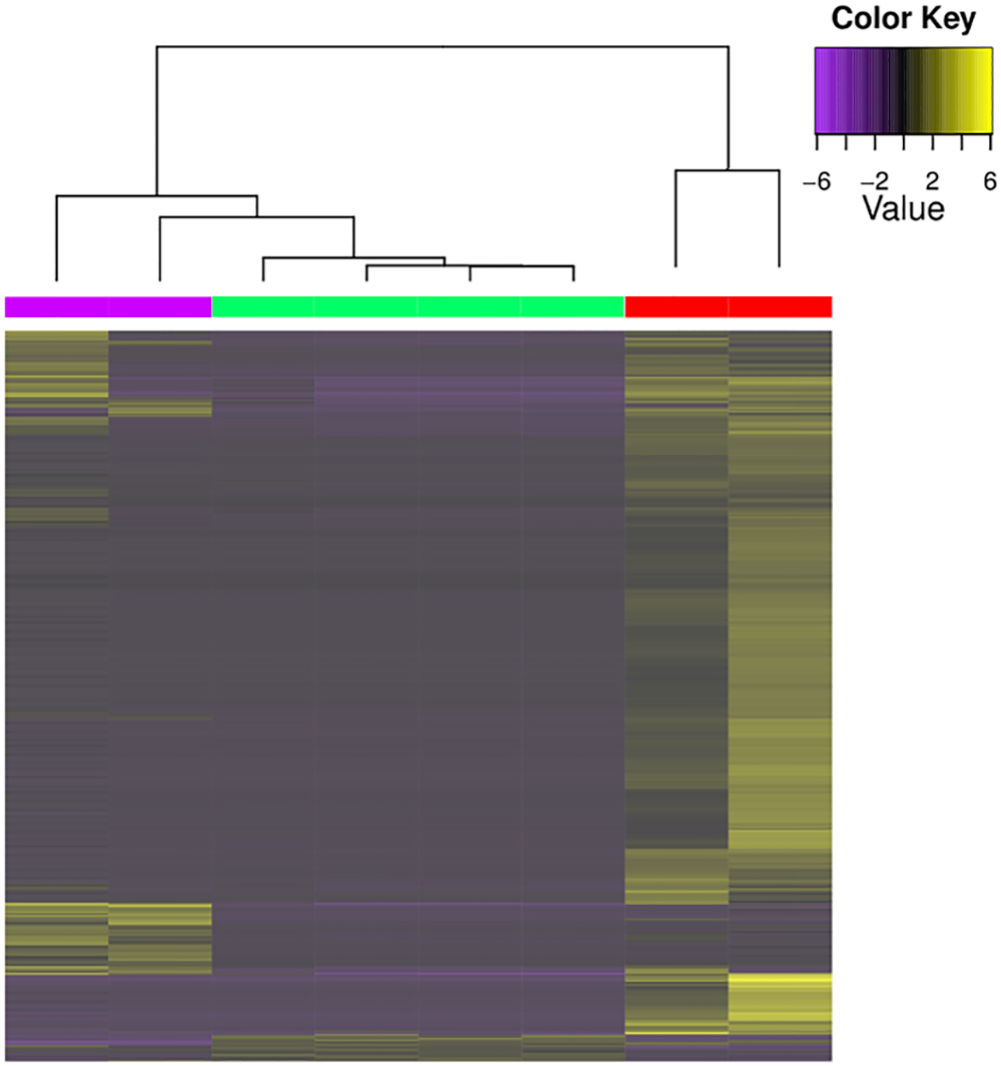
Heatmap showing differential expresssion across host plants. (Purple: *Tetrazygia bicolor*, Green: *Psychotria nervosa*, Red/: *Myrsine cubana*). Heatmap colors correspond to log2-transformed RPKM (Fragments Per Kilobase of transcript per Million mapped reads) values for each transcript. Yellow genes are upregulated in at least one pairwise comparison between the three host plants and purple genes are downregulated.

We identified 2,028 transcripts as differentially expressed across all comparisons. Most differentially expressed transcripts were upregulated on *M*. *cubana* relative to the other two plant species, but 280 and 58 genes were upregulated in *T*. *bicolor* and *P*. *nervosa* respectively (Fig 1). Only 23 transcripts matching detoxification genes were significantly differentially expressed: 3 UDP glycosyltransferases, 4 cytochrome P450s, 2 glutathione S-transferases, 1 glutathione peroxidase, and 13 subunits of ABC transporters. Each was upregulated in *M*. *cubana*, except for 2 ABC transporter subunit transcripts and 1 glutathione S-transferase, which were upregulated in *T*. *bicolor* compared to *P*. *nervosa*. Of the 1015 transcripts which closely resemble possible aphid effectors, 8 were significantly differentially expressed (Table 2). Seven effector homologs were upregulated on *M*. *cubana* and one on *T*. *bicolor*.

**Table 2:** Differentially expressed aphid effector homologs and what is known about their function.

Comparison & Upregulation	Contig ID	Effector Blast Hit	Protein Name	Mode Of Action	References
***P*. *nervosa* vs *T*. *bicolor***					
Upregulated on *T*. *bicolor*	c60978_g1_i1	ACYPI009755-RA	Disulfide isomerase	Increases salivary protein formation in nematodes	Geldhof et al. (2003)[39]
***M*. *cubana* vs *P*. *nervosa***					
Upregulated on *M*. *cubana*	c29924_g1_i1	ACYPI002622-RA	Calreticulin	May circumvent calcium-mediated wound responses of host plant, prevents sieve tube occlusion	Carolan et al. (2011)[10]
Upregulated on *M*. *cubana*	c38772_g1_i1	ACYPI008001-RA	ARMET/Endopeptidase inhibitor	Found in pea aphid saliva to assist aphid feeding	Wang et al. (2015)[40]
Upregulated on *M*. *cubana*	c38738_g1_i1	ACYPI003917RA	SCP GAPR-1	Similar to plant pathenogenesis protein (PR-1), alters defense mechanisms	Carolan et al. (2009)[41]
Upregulated on *M*. *cubana*	c94746_g1_i1	ACYPI008370-RA	CLIP-domain serine protease	Inhibits phenol oxidase-based innate defenses of plants	Carolan et al. (2011)
Upregulated on *M*. *cubana*	c37751_g1_i1	ACYPI009427-RA	M1 zinc metalloprotease	Deactivation of plants defense signaling peptides and dietary plant protease inhibitors in insect gut	Carolan et al. (2011)
Upregulated on *M*. *cubana*	c30871_g1_i1	ACYPI000288-RA	Glucose dehydrogenase	Suppresses plant defense mechanism	Nicholson et al (2012)[42]
Upregulated on *M*. *cubana*	c41723_g1_i1	Gi|109195254|gb| EC388700.1 |EC388700	Retinol dehydrogenase	Binds retinols and fatty acids and has been described to bind to lipid jasmonate precursors in *M*. *javanica*	Iberkleid et al. (2013)[43]

These homologs are significantly upregulated on one host plant in comparison to their average expression level on the other host plant. Host plant comparisons are in bold.

### Functional enrichment analysis

The genes upregulated in lobate lac scale feeding on *M*. *cubana* were enriched for GO terms related to functions such as ion transport, ATP hydrolysis-coupled proton transport, regulation of gene expression, GTP binding, oxidoreductase activity on NADPH, phosphatase activity, ion binding, and metalloendopeptidase activity (Table 3). In contrast, on *P*. *nervosa*, upregulated expression was significantly enriched for macromolecule localization and protein catabolism. Enriched GO terms on *T*. *bicolor* included actomyosin structure, cellular component organization, and regulation of gene expression.

**Table 3:** Functional enrichment analysis.

Host Plant Comparison	Upregulated Genes	Top Enriched Biological Processes	Top Enriched Molecular Functions
*Myrsine cubana* vs *Psychotria nervosa*	*M*. *cubana* 1738 *P*. *nervosa* 57	*M*. *cubana* **Membrane fusion** **Ion transport** Cellular protein modification *P*. *nervosa*: Ubiquitin-dependent protein catabolism Macromolecule localization	*M*. *cubana* GTP binding Oxidoreductase activity on NADPH Phosphoprotein phosphatase activity **Phospholipid binding** Endopeptidase inhibitor activity
*Myrsine cubana* vs *Tetrazygia bicolor*	*M*. *cubana* 1521 *T*. *bicolor* 195	*M*. *cubana* Post-transcriptional regulation of gene expression ATP hydrolysis coupled proton transport Response to external stimulus *T*. *bicolor* Actomyosin structure organization Gene silencing by RNA	*M*. *cubana* Protein binding Metalloendopeptidase activity **Ion binding** Zinc binding **Calcium binding** *T*. *bicolor* Structural molecule activity
*Psychotria nervosa* vs *Tetrazygia bicolor*	*P*. *nervosa* 36 *T*. *bicolor* 246	*P*. *nervosa* Macromolecule localization *T*. *bicolor* Negative regulation of gene expression Cellular component organization	*P*. *nervosa* Translation factor activity, RNA binding *T*. *bicolor* Motor activity ATP binding

Enriched GO terms among genes significantly upregulated on a host plant species relative to another in a pairwise comparison. GO terms potentially related to detoxification are italicized. GO terms that are potentially related to effector activity are in bold. Terms related to primary metabolism are underlined.

Many of the GO terms enriched for differential expression on *M*. *cubana* are thought to be involved in detoxification (Table 3). Specific mechanisms are not clear, but increased expression of these genes has been associated with exposure to plant chemical defenses [44]. By contrast, these GO terms are not enriched on *T*. *bicolor* and *P*. *nervosa*. Instead, on those host plant species, we saw increased expression of genes involved in primary metabolism.

## Discussion

The recent comparative genomics work is beginning to show us general features of the genetic architecture of host-use variation in plant-eating insects. In this study, we find additional support for the importance of host-dependent expression of detoxification genes. We also find further evidence that detoxification genes account for only a small part of all host-induced gene expression changes, and that there is a trade-off between expression of genes for detoxification and those for primary metabolism. We recover evidence of a striking diversity of effector proteins in lobate lac scale; more than a thousand transcripts, ~ 1% of all transcripts in the sample, are near matches to one of 87 putative aphid effectors, and several of these transcripts are differentially expressed in the lobate lac scale across host plant species. Nevertheless, effectors as a group were not enriched for differential expression; most of them are expressed at the same level across hosts.

### Detoxification genes

Of the 1196 putative detoxification genes that were expressed, only 23 were significantly upregulated on any one host. Pervasive host-insensitivity in the expression of detoxification genes appears to be the rule across plant-eating insects [18, 45]. However, we did find that ABC transporters were more differentially expressed across hosts than expected by chance. The pool of differentially expressed genes was significantly enriched for genes involved in ATP hydrolysis-coupled proton transport (p-value = 1.6e-5) (Table 3), a process carried out by ATPase proteins, which are subunits of ABC transporter proteins. ABC transporter expression is associated with xenobiotic elimination and insecticide resistance in several insect species [44].

Only a few of the genes that we identified *a priori* as being involved in detoxification were found to be differentially expressed, but perhaps our *a priori* assignments were too exclusive. In fact, some of the enriched GO terms in lobate lacs scale from *M*. *cubana* could be indicative of host-induced differences in the expression of genes involved in detoxification pathways (Table 3). Most notably, oxidoreductase activity on NADPH is often linked to detoxification, as several detoxification proteins use NADPH as an electron acceptor [46]. These proteins include cytochrome P450 oxygenases, and glutathione peroxidases [47]. In other words, we found significantly enriched differential expression of some genes that are adjacent to detoxification genes in metabolic networks. Furthermore, oxidoreductase activity and zinc binding, other enriched GO terms, are functions of zinc-binding dehydrogenases, which appear to be important for plant defense compensation in peach aphids [12]. Thus, if we consider a more inclusive set of genes involved in plant-defense compensation, our expression data provides more support for the importance of host-induced differential expression.

### Aphid effectors on *M*. *cubana*

Each of the differentially expressed effector transcripts is likely to have a similar function to its closest matching aphid transcript (Table 2). These effectors have several functions. M1 zinc metalloprotease is one of the most well-understood effector proteins. Sap-sucking insects use it to destroy plant signaling defense peptides and improve phloem sap quality by increasing free amino acid content [10]. A second effector that we know something about is calreticulin. Calcium ions transmit information in plant signaling pathways. Calreticulin binds calcium ions and disrupts these signals. This prevents plants from closing off compromised sieve tube elements, which would cut off the supply of phloem sap [10]. The over-expression of a calreticulin homolog may account for the enrichment of calcium binding processes we found in lobate lac scale on *M*. *cubana*. A third effector with characterized function is retinol dehydrogenase. It attracts retinols and fatty acids and can bind lipid jasmonate precursors to prevent jasmonate pathway defenses [43]. This may relate to the enriched lipid and phospholipid binding we observe in lobate lac scale on *M*. *cubana*.

### Induction of effectors and detoxification genes in *M*. *cubana*

The great majority of differentially expressed genes were upregulated on *M*. *cubana*. This could indicate that *M*. *cubana* is simply better defended than the other plant species. Plants in the Myrsinaceae family are known to produce a variety of benzoquinone compounds, characterized by an aromatic ring and two carbonyl groups [48]. Benzoquinones and their derivatives have antibacterial, insecticidal, and anti-feeding properties [49]. Benzoquinones are toxic to several species of aphid as well as the extremely polyphagous red spider mite, which shows that they could be difficult to process by generalist insect species [50]. Many species of Myrsinaceae also accumulate saponins in their tissues. Saponins are cytotoxic chemicals composed of a hydrophobic aglycone (non-sugar) attached to glycoside (sugar residue) [48]. They play an important role in plant defense against insects and parasites, and they also elicit immune responses in people [51]. Myrsinaceous plants can also be induced to produce proteinase inhibitors known as phytostatins in response to wounding [52]. These proteinase inhibitors make their way to the insect gut and inhibit digestion. In comparison to *M*. *cubana*, *P*. *nervosa* and *T*. *bicolor* are thought to be less protected by defensive chemistry [53, 54]

### DE of gene expression regulation

We found that some of the most conspicuous changes in gene expression across hosts occurs at genes involved in regulating gene expression. In lobate lac scale on *T*. *bicolor*, we found enrichment of genes for gene silencing by RNA. This was previously shown on some hosts of *Myzus persicae* (in which case miRNA activity is negatively correlated with the abundance of a P450 enzyme) [55]. In lobate lac scales on *P*. *nervosa*, we found enriched expression of genes for RNA binding and translation factor activity, both of which are thought to play a role in eukaryotic post-transcriptional regulation [56]. Previous RNA-seq studies of how gene expression differs across hosts of plant-eating insect species have also found differential expression of genes for ribosomal proteins and nucleic acid binding. This kind of differential expression may integrate across GO categories and be indicative of broad-scale expression changes that may nonetheless not amount to significant enrichment of differential expression within GO categories.

### Caveats

In this study, some of the gene expression variation that we observed across hosts could be due to biotic and abiotic factors that covary with hosts across sites. For example, gene expression variation could correspond to variation in local light and soil conditions, or assemblages of other plant-eating insects and natural enemies. In future studies, we could minimize the noise caused by such factors by using common garden experimental designs, or sampling much more intensively across wild populations. The strictly bioinformatics approach that we have taken to functional analysis also has limitations. The function of candidate genes for host-generalism could be more accurately characterized with genome editing approaches such as RNA interference. And that may yield insights that could yield new tools to control populations of pests.

## Conclusion

For decades, we assumed that host use in a plant-eating insect is limited by their capacity to cope with the chemical defenses of plant species, but we knew little about the details. In the last few years, comparative transcriptomics analyses of plant-eating insects have identified hundreds of genes that are differentially expressed across hosts, including many genes suspected to be involved in the detoxification of plant chemicals. They have also shown us that host-use adaptation is much more complex than activating or silencing the expression of a few detoxification genes, and may entail gene expression trade-offs between plant-defense compensation and primary metabolism. Here, by analyzing gene expression plasticity across three host species of the lobate lac scale insect, we find further support for each of these insights. Moreover, we find evidence that the genes that are directly involved plant-defense-compensation may vary across plant-eating insect species, and may be more diverse than commonly thought. We also find evidence that in some cases, host-defense compensation may depend on differential expression of genes that are adjacent to detoxification enzymes in metabolic pathways. These insights have the potential to pave the way for a more useful theory of host-use adaptation, and for new, more economical and sustainable tools for managing pest insect populations.
